# Impact of *Schistosoma japonicum* Infection on Collagen-Induced Arthritis in DBA/1 Mice: A Murine Model of Human Rheumatoid Arthritis

**DOI:** 10.1371/journal.pone.0023453

**Published:** 2011-08-08

**Authors:** Xiaorong Song, Jilong Shen, Huiqin Wen, Zhengrong Zhong, Qinli Luo, Deyong Chu, Yao Qi, Yuanhong Xu, Wei Wei

**Affiliations:** 1 Institute of Clinical Pharmacology, Anhui Medical University, Hefei, Anhui, China; 2 The Key Laboratory of Microbiology and Parasitology, and The Key Laboratory of Zoonoses, Anhui Medical University, Hefei, Anhui, China; 3 Institute of Anhui Cardiovascular Research, Anhui Provincial Hospital, Hefei, Anhui, China; University Hospital Freiburg, Germany

## Abstract

**Background:**

The hygiene hypothesis suggests that helminth infections prevent a range of autoimmune diseases.

**Methodology/Principal Findings:**

To investigate the effects of *S. japonicum* infection on collagen-induced arthritis (CIA), male DBA/1 mice were challenged with unisexual or bisexual *S. japonicum* cercariae two weeks prior to bovine type II collagen (CII) immunization or at the onset of CIA. *S. japonicum* infection prior to CII immunization significantly reduced the severity of CIA. ELISA (enzyme linked immunosorbent assay) showed that the levels of anti-CII IgG and IgG2a were reduced in prior schistosome-infected mice, while anti-CII IgG1 was elevated. Splenocyte proliferation against both polyclonal and antigen-specific stimuli was reduced by prior schistosome infection as measured by tritiated thymidine incorporation (^3^H-TdR). Cytokine profiles and CD4^+^ T cells subpopulation analysis by ELISA and flow cytometry (FCM) demonstrated that prior schistosome infection resulted in a significant down-regulation of pro-inflammatory cytokines (IFN-*γ*, TNF-*α*, IL-1*β* and IL-6) and Th1 cells, together with up-regulation of the anti-inflammatory cytokine IL-10 and Th2 cells. Interestingly, the expansion of Treg cells and the reduction of Th17 cells were only observed in bisexually infected mice. In addition, prior schistosome infection notably reduced the expression of pro-inflammatory cytokines and receptor activator of NF-κB ligand (RANKL) in the inflamed joint. However, the disease was exacerbated at one week after infection when established CIA mice were challenged with bisexual cercariae.

**Conclusion/Significance:**

Our data provide direct evidence that the Th2 response evoked by prior *S. japonicum* infection can suppress the Th1 response and pro-inflammatory mediator and that bisexual infection with egg-laying up-regulates the Treg response and down-regulates the Th17 response, resulting in an amelioration of autoimmune arthritis. The beneficial effects might depend on the establishment of a Th2-dominant response rather than the presence of the eggs. Our results suggest that anti-inflammatory molecules from the parasite could treat autoimmune diseases.

## Introduction

Helminth parasites are prevalent in humans, especially in tropical and subtropical areas [1]. Chronic infections are characterized by a Th2-dominant response as well as an overall down-regulated immune system [1], [2]. This helminth-induced immunosuppression may spill over to un-related antigens, down-regulate the response to other pathogens. Recent studies have suggested that helminth infection is protective in murine models of autoimmune disorders and asthma [3]. Nematodes have been used to effectively treat human inflammatory bowel disease (IBD) [4], [5], [6].

Rheumatoid arthritis (RA) is an autoimmune disease of unknown etiology that afflicts about 1% of the population [7]. In addition to disability and decreased quality of life, RA also decreases life expectancy due to accelerated atherosclerosis. Therapies of RA vary from conventional disease-modifying anti-rheumatic drugs (DMARDs) to biologics. The introduction of novel biologics in the 1990s notably improved clinical outcomes in RA. Cytokine antagonists that inhibit TNF-*α*, IL-6 or IL-1*β* decrease inflammation and joint destruction [8], [9]. Unfortunately, these therapies are only effective in about half of patients, and therapies targeting cytokines can interfere with immune defense [8]. Therefore, there is still a need for the identification of new pathways involved in the modulation of inflammation to improve the inhibition of autoimmune responses while maintaining an effective response to infectious agents.

Classically, RA was thought to be mediated by the Th1 response. Th1 cells are enriched in synovial tissue, where they release IFN-*γ*, TNF-*α* and lymphotoxin *β*, which aggravate joint inflammation [10], [11]. Th1 lineage specific transcription factors, T cell-specific transcription factors (T-bet), signal transducer and activator of transcription 4 (STAT4) and its promoting cytokine IL-12 are required for the development of the autoimmune disorder [9], [10].

Our immune system has been co-evolving with parasites for millions of years. The ability of helminth to survive in their hosts is due to their potential to maintain a balance of immune activation and suppression in the host [12]. Recent studies showed that inoculation with helminths or helminth-derived products markedly reduced the clinical manifestations of experimental arthritis [13], [14], [15]. However, the mechanism of this beneficial effect remains undefined. Schistosome infection can modify the phenotype and function of DC, Mϕ, NKT and B1 B cells, leading to the expansion of Th2 and Treg populations that might be responsible for maintaining self-tolerance [12], [16], [17]. Recent data have demonstrated that the newly found CD4^+^ T cell subset Th17 and its signature cytokine IL-17 might be involved in *S. mansoni*-induced suppression of autoimmune and allergic disorders [15], [18], [19].

According to classical Th1/Th2 dichotomy, we speculate that Th2-biased response induced by *S. japonicum* infection could ameliorate Th1 mediated CIA in DBA/1 mice—a traditional animal model of human RA. Although Th2 response develops with the onset of egg produced by female worm in full-blown infection, it has also been reported in larvae stage infection and unisex cercariae infection, where no egg laying takes place. In this work, we try to characterize the immunomodulatory effects of both uni- and bi-sexual *S. japonicum* infection on autoimmune arthritis. Possible anti-inflammatory mechanisms are examined by studying CD4^+^ T helper cell subpopulations and the cytokine expression profiles of the CIA mice infected by schistosomes.

## Results

### Prior *S. japonicum* infection significantly attenuates clinical signs of CIA

**Figure 1:**
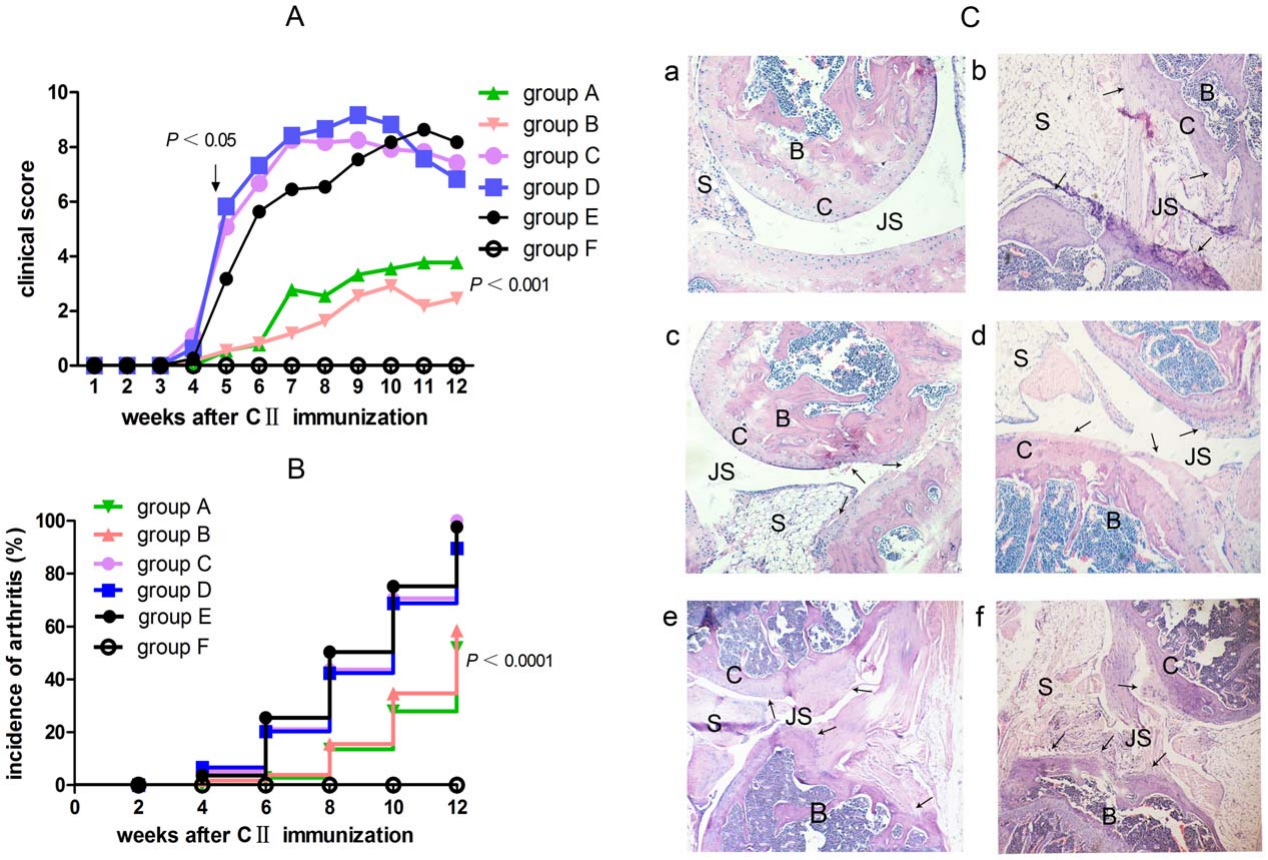
Effects of *S. japonicum* infection on the development of collagen-induced arthritis (CIA) in DBA/1 mice. Attenuated clinical manifestation of arthritis in prior *S. japonicum* infected mice. Mice were infected with *S. japonicum* 2 weeks prior to bovine type II collagen (CII) immunization or at the onset of disease. The severity (A) and incidence (B) of arthritis were assessed as described in Materials and Methods. Data are expressed as means of the total scores for four limbs or the incidence of arthritis after CII immunization. (n = 12/group). Statistical analysis by one-way ANOVA with Bonferroni's multiple comparison tests or by log-rank test of Kaplan–Meier survival curves. The clinical score of each mouse was recorded every day from 14 d after primary CII immunization. The onset of arthritis was about 28 d after CII immunization, and the peak time of the onset was around 33–35 d after immunization. The clinical score and the survival curve only differ significantly between prior schistosome-infected CIA mice and uninfected control mice. The diseases were exacerbated in the established CIA mice at one week after bisexual cercariae challenging. group A = unisexual *S. japonicum* infection prior to CII immunization; group B = bisexual *S. japonicum* infection prior to CII immunization; group C = unisexual *S. japonicum* infection post CII immunization; group D = bisexual *S. japonicum* infection post CII immunization; group E = uninfected CIA control mice, group E = age-matched normal control mice. (C) (a), A normal mouse and (b) a Type-II collagen (CII)-immunized uninfected mouse. Note the presence of severe synovial hyperplasia, mononuclear cell infiltration and angiogenesis, and the destruction of cartilage, focal fibrotic ankylosis and stenosis of articular cavity in the CII-immunized uninfected mouse. The arrows indicate an area of cartilage and bone (calcaneus) destruction. (c and d), Prior unisexual and bisexual *S. japonicum* infected CIA mouse. (e and f), Unisexual and bisexual *S. japonicum* infected in an established CIA mouse. Note the marked reduction of cell influx. Neither severe synovitis nor synovial hyperplasia was observed. Abbreviations: *S. japonicum*, *Schistosoma japonicum*; CII, bovine Type-II collagen; B, bone (calcaneus); JS, joint space; C, cartilage; S, synovitis. Original magnification: 100×.

DBA/1 mice developed signs of arthritis around 4 weeks after CII immunization. Both unisexual and bisexual infection with *S. japonicum* prior to CII immunization markedly reduced the arthritis score (Figure 1A) and the incidence of arthritis (Fig. 1B). The inhibitory effects of schistosome infection on CIA were not correlated with unisexual versus bisexual *S. japonicum* infection. However, when the established CIA mice (4 weeks after the first CII immunization) were infected with bisexual *S. japonicum*, the severity of arthritis was remarkably aggravated at one week after *S. japonicum* infection.

### Prior *S. japonicum* infection reduces CIA histological damage in DBA/1 mice

Figure 1C (a) shows the histopathology of knee joints from normal mice, (b) uninfected CIA mice, (c, d) prior unisexual or bisexual *S. japonicum*-infected CIA mice, and (e, f) post unisexual or bisexual *S. japonicum*-infected CIA mice. Prior *S. japonicum* infection ameliorated the synovial hyperplasia, mononuclear cell infiltration and angiogenesis in the inflamed synovium. The destruction of cartilage, focal fibrotic ankylosis and stenosis of articular cavity were also alleviated in prior schistosome-infected CIA mice. No pronounced improvement of histopathology was observed in the post-infected CIA mice. The uninfected CIA mice showed remarkable synovial hyperplasia, inflammatory cell influx, angiogenesis and destruction of cartilage and bone.

### Prior *S. japonicum* infection alters the humoral immune response in CIA mice

**Figure 2:**
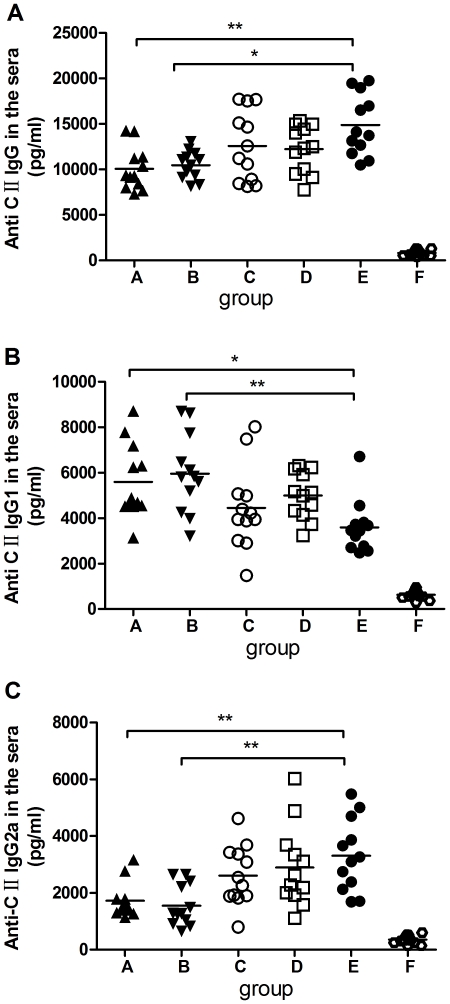
Effects of *S. japonicum* infection on anti-type-II collagen (anti-CII) antibody production. *S. japonicum* infection alters the levels of auto-antibodies in CIA mice. Sera were taken from DBA/1 mice 12 weeks after CII immunization. The concentration of anti-CII IgG and its subtype antibodies were determined by ELISA. The levels of anti-CII IgG and its subtypes were elevated in CIA mice. Compared with uninfected CIA mice, prior schistosome infection significantly reduced the levels of IgG and IgG2a in the sera, while the level of IgG1 was elevated in the infected mice. However, there was no statistically significant difference in the post schistosome infected CIA mice. No difference was observed between unisexual and bisexual infected mice. Means ± SD data for each mouse are shown. Compared with CIA control group, *: *P*<0.05; **: *P*<0.01. Data are representative of three repeated experiments. Abbreviations: ELISA, enzyme linked immunosorbent assay. See Figure 1 for other definitions.

To determine the influence of schistosome infection on the humoral anti-collagen response, we compared the levels of anti-collagen IgG and its subclasses in the sera. As shown in Figure 2A, a significantly decreased level of anti-CII IgG was present in prior schistosome-infected CIA mice compared with uninfected controls. The levels of anti-CII IgG1 and IgG2a serve as extremely valuable in vivo markers of Th2 and Th1 responses, respectively [20]. We further compared the levels of IgG1 and IgG2a subclasses in prior and post-infected CIA mice with those of uninfected CIA animals. The level of anti-CII IgG1 (Figure 2B) was significantly elevated and that of anti-CII IgG2a (Figure 2C) was reduced in prior infection CIA mice compared with uninfected CIA mice. No significant differences in the levels of anti-CII IgG and its subclasses were observed between unisexual and bisexual schistosome-infected CIA mice.

### Prior *S. japonicum* infection inhibits splenocyte proliferation in CIA mice

**Figure 3:**
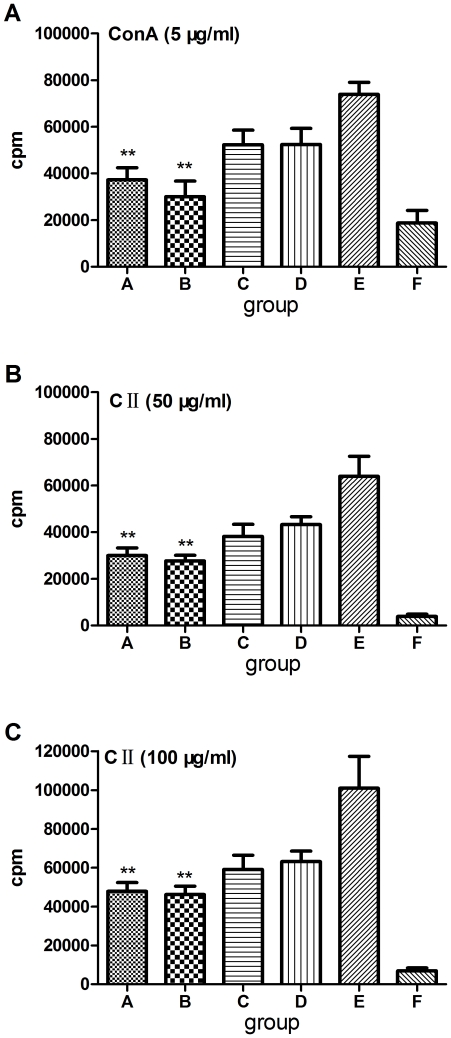
Effects of *S. japonicum* infection on splenocyte proliferation. *S. japonicum* infection inhibited splenocyte proliferation *in vitro*. 12 weeks after CII immunization in DBA/1 mice, splenocytes were cultured in RPMI 1640 containing 10% fetal calf serum for 72 hrs in the presence of polyclonal (ConA 5 µg/ml) or antigen-specific stimuli (CII 50 µg/ml or 100 µg/ml), respectively. Splenocyte proliferation was analyzed by ^3^H-TdR. Proliferation data are presented as mean ± SE of c.p.m. values. Data are representative of three repeated experiments. Compared with CIA control group, *: *P*<0.05; **: *P*<0.001. Abbreviations: ^3^H-TdR, tritiated thymidin incorporation; c.p.m., counts per minute. See Figure 1 for other definitions.

We assessed the effect of infection with *S. japonicum* on the T cell proliferative response to polyclonal or antigen-specific stimuli by tritiated thymidine incorporation. In contrast to prior *S. japonicum*-infected CIA mice, splenocytes from the uninfected CIA control group displayed strong proliferative responses to ConA (Figure 3A) and bovine CII (Figure 3B and 3C). No notable differences were observed between mice infected with unisexual and bisexual cercariae.

### Prior *S. japonicum* infection promotes a shift in cytokine production from pro-inflammatory to anti-inflammatory

**Figure 4:**
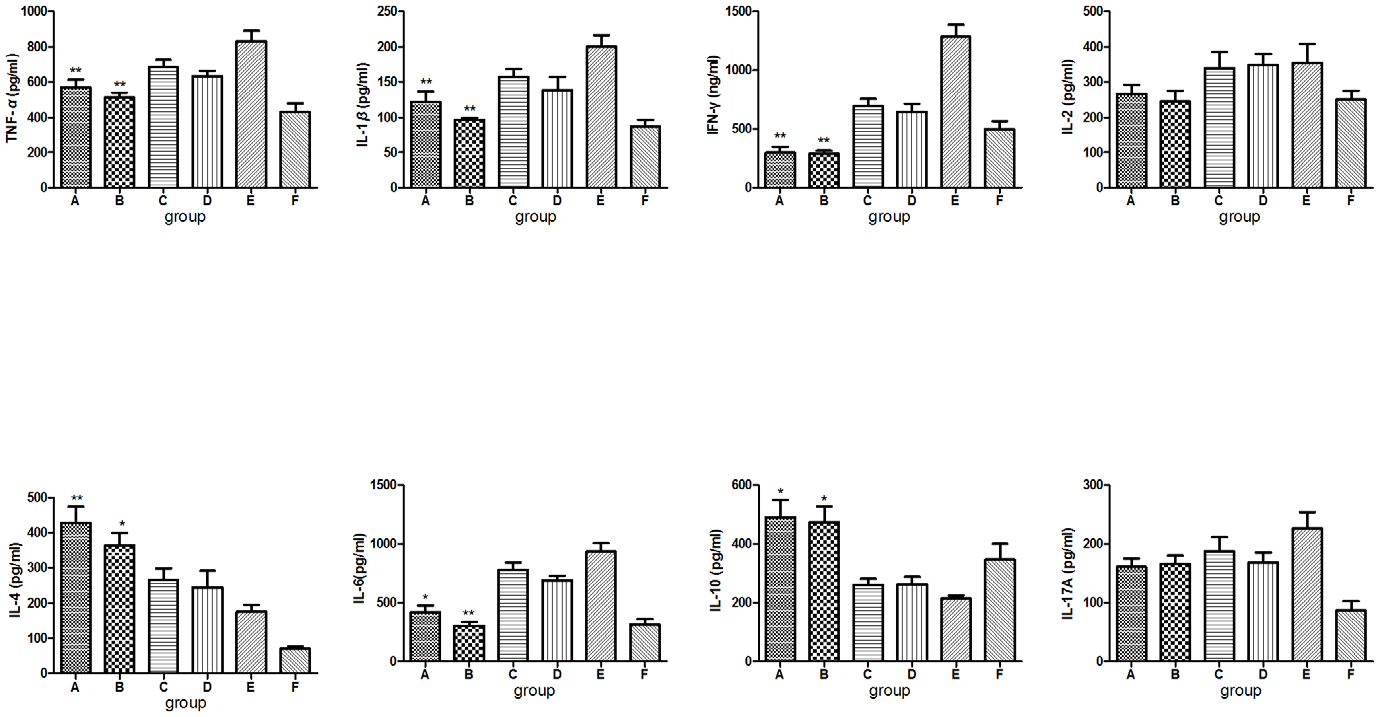
*S. japonicum* infection induced a shift in systemic cytokine profiles from pro-inflammation to anti-inflammation. *S. japonicum* infection induced a shift in cytokine patterns from pro-inflammatory to anti-inflammatory. Splenocytes obtained from DBA/1 mice 12 weeks after CII immunization were cultured *in vitro* in RPMI 1640 containing 10% fetal calf serum with ConA (5 µg/ml) for 48 hrs. Supernatants were collected and analyzed for cytokine production by ELISA as described in Materials and Methods. Note that the levels of the Th1 signature cytokine IFN-*γ* and the pro-inflammatory cytokines TNF-*α*, IL-1, IL-6 were lowered, while those of the Th2 signature cytokine IL-4 and IL-10 were enhanced in prior *S. japonicum*-infected mice. No differences were found between unisexual and bisexual infected mice. Data are expressed as means ± SD for duplicate cultures. Compared with CIA control group, *: *P*<0.05; **: *P*<0.01. See Figures 1 and 2 for other definitions.

To determine the systemic influence of *S. japonicum* infection on cytokine production potential in schistosome-infected CIA mice and on those from uninfected mice, we analyzed cytokines in splenocyte culture supernatants using ELISA. As shown in Figure 4, our data revealed that IFN-*γ* production was decreased in prior *S. japonicum*-infected mice whereas IL-4 production was enhanced, suggesting an apparent Th2-dominant response. Moreover, we also observed decreases in the levels of TNF-*α*, IL-1*β*, IL-6 and an increase in the level of IL-10 in the culture supernatant of splenocytes in prior *S. japonicum*-infected mice. No differences were observed in the production of IL-2 and IL-17A between infected and uninfected mice. No difference was observed between mice infected with unisexual and bisexual infected mice.

### Protective effects of prior schistosome infection were associated with enhanced systemic Th2 response, but the expansion of CD4^+^CD25^high^ Tcells were only induced by bisexual infection

**Figure 5:**
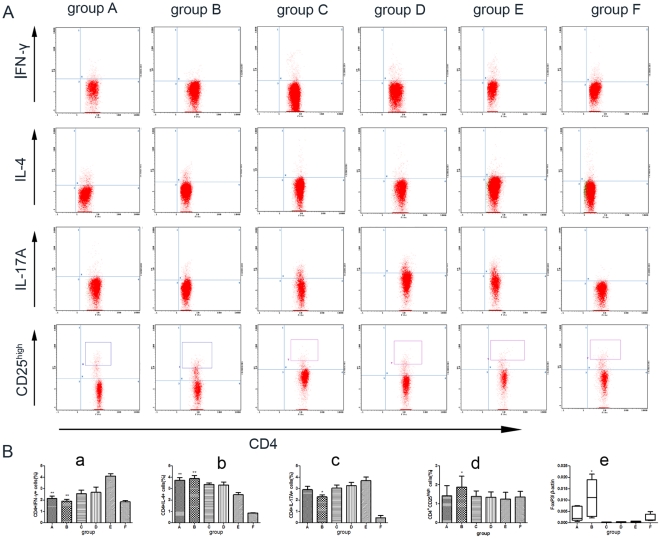
The protective effects of *S. japonicum* infection were associated with expanding Th2/Treg cell subpopulations. A and B (a, b, c, d), Elevated Th2/Treg cells in prior infection mice. Splenocytes were obtained from DBA/1 mice 12 weeks after CII immunization. Splenocytes were analyzed by FCM for CD4^+^CD25^high^ Tcells, or CD4^+^IFN-*γ* ^+^, CD4^+^IL-4^+^ and CD4^+^IL-17A^+^ cells after *in vitro* re-stimulation with PMA/ionomycin in the presence of monesin for 4 hrs (See Materials and Methods for detail). Both unisexual and bisexual infection prior CII immunization enhanced the percentage of CD4^+^IL-4^+^ T cells and reduced the presence CD4^+^IFN-γ^+^ T cells in the spleen. However, the increase in CD4^+^CD25^high^ Tcells and the decrease in CD4^+^IL-17A^+^ T cells were only noted in bisexually-infected mice. B (e), the elevated mRNA expression of Foxp3 was also demonstrated only in prior bisexually-infected mice. Compared with CIA control group, *: *P*<0.05; **: *P*<0.001. Abbreviations: FCM, flow cytometry; Foxp3, foxhead box protein 3. See Figure 1 for other definitions.

As shown in Figure 5A and 5B, the percentage of Th2 cells was elevated in prior unisexual and bisexual infected mice while the percentage of the Th1 population was reduced compared with uninfected CIA mice. Interestingly, an increase in the percentage of CD4^+^CD25^high^ T cells and a decrease in Th17 cells were only observed in prior bisexual schistosome-infected mice. Real-time PCR also demonstrated an increase in foxhead box protein 3 (Foxp3) mRNA expression in splenocytes only in prior bisexual schistosome-infected CIA mice. Our data also demonstrated that the total numbers of slenocytes were increased significantly in bisexually schistosome-infected mice (2.04±0.28×10^8^, 2.07±0.46×10^8^ in prior and post bisexually infected CIA mice vs 0.48±0.17×10^8^, 0.53±0.17×10^8^, 0.48±0.13×10^8^, 0.45±0.12×10^8^ in prior and post unisexually infected CIA mice, CIA control mice, normal control mice, respectively, *P*<0.001). The absolute number of each Th subset was increased correspondingly. No difference was found among unisexually infected CIA mice, CIA control mice and age-matched normal control mice.

### *S. japonicum* infection attenuates the augmentation of inflammatory mediators in the inflamed joint

**Figure 6:**
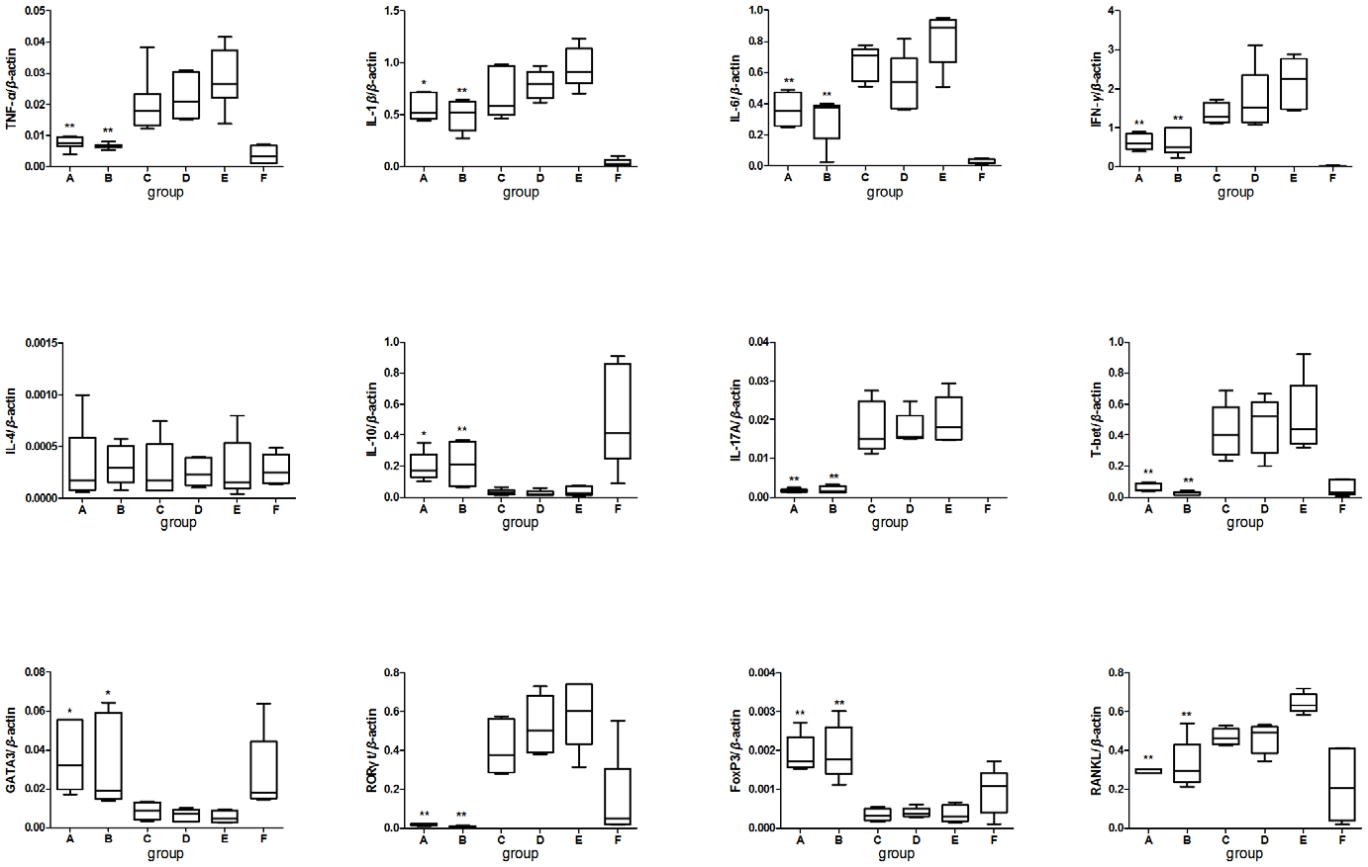
Prior *S. japonicum* infection reduced the expression of immune mediators in inflammatory joints. Reduced pro-inflammatory cytokines expression in the focal joints in *S. japonicum* infected mice. The mRNA expression of cytokines and RANKL were quantified by Real time PCR as described in Materials and Methods. Prior *S. japonicum infection balanced the pro- and anti-inflammatory cytokines and reduced the expression of RANKL in the local site. Values along the vertical axis represent relative expression levels normalized to β*-actin. Compared with CIA control group, *: *P*<0.05; **: *P*<0.001. Data are representative of three repeated experiments. Abbreviations: RANKL, receptor activator of NF-κB ligand. See Figure 1 for other definitions.

Cytokine profiles at the immediate sites of inflammation are believed to differ significantly from those in systemic lymphatic tissue [21]. Therefore, we examined the gene expression of pro/anti-inflammatory cytokines (TNF-*α*, IL-1*β*, IL-6 and IL-10), the signature cytokine and specific transcription factor of Th cell subpopulations, and RANKL in the joints. As shown in Figure 6, a decrease in pro-inflammatory cytokines TNF-*α*, IL-1*β*, IL-6 and an increase in IL-10 were noted in schistosome-infected CIA mice compared with uninfected mice, with a statistically significant difference observed in prior infected mice. Examining the signature cytokines of Th cell subpopulations and their key transcription factors, the levels of IL-17A, IFN-*γ*, T-bet and retinoic orphan receptor γt (RORγt) were down-regulated by prior schistosome infection, while GATA binding protein-3 (GATA3) and Foxp3 were up-regulated. We also noted a significant reduction in RANKL, an essential activator for osteoclast development, in prior schistosome-infected CIA mice. Unexpectedly, no enhancements of IL-4 were observed in schistosome-infected CIA mice.

## Discussion

In this study, we have experimentally investigated the immunomodulatory influence of *S. japonicum* unisexual (worm only) and bisexual (worm+eggs) infection on CIA mice. We have demonstrated both the protective and the deteriorative roles of schistosome infections in a murine model of arthritis. We confirmed the prophylactic effects of schistosome infection by showing that both unisexual and bisexual *S. japonicum* cercariae challenge 2 weeks prior to induction of CIA suppressed CIA as indicated by the reduction of the arthritis score and the incidence at 12 weeks post CII immunization. The alleviation of the chronic synovitis seen at histopathology and the prevention of damage to the articular cartilage indicated that *S. japonicum* infection also has an inhibitory effect on arthritis at the structural level. No differences were observed between unisexual and bisexual infection in clinical manifestation and the incidence of arthritis in prior schistosome-infected CIA mice.

The development of Th cells in bisexual schistosome-infected mice is clearly consistent, while that in unisexual infection is poorly defined. It is believed that mice develop a Th1 response during the first 5–6 weeks of infection. As the infection progresses, a Th2 response develops and Th1 wanes [1], [22]. In our experiment, we observed that in the early phase (before 6 weeks of schistosome infection), *S. japonicum* infection exacerbated the severity of arthritis when established CIA mice were infected with both unisexual and bisexual schistosome, with these findings reaching statistical significance in bisexually-infected mice at one week after schistosome infection. The deteriorative effects of bisexual schistosome infection in CIA mice could be due to the vigorous Th1 response induced by bisexual infection in a pro-inflammatory environment, while the mechanism of action of unisexual infection remains to be elucidated. Interestingly, we also noted that *S. japonicum* infection resulted in symptom relief in post schistosome infected-CIA mice 6 weeks after infection although these findings were not statistically significant. Thus, we presumed that the beneficial effects in prior schistosome-infected mice may depend on the prior establishment of a Th2-dominant response, making the host unable to initiate a pathogenic Th1 response in the host, rather than depending on the presence of eggs. Even in the established disease, the Th2 response elicited by *S. japonicum* infection worked towards relieving the clinical signs of arthritis, implying a therapeutic effect of the Th2 response in CIA.

The anti-CII antibodies have been recognized as important pathogenic factors in the initiation and development of CIA [20], [23]. As the Th1 response is expected to be down-regulated in *S. japonicum*-infected mice, we measured the levels of total IgG and its subclasses IgG1 and IgG2a, which are specific for bovine CII. At 12 weeks post-immunization, *S. japonicum* infection resulted in decreased levels of IgG and IgG2a but increased IgG1 in prior schistosome-infected CIA mice when compared with the uninfected control group. This alteration in class switch may be at least partially related to the amelioration of arthritis by *S. japonicum* infection.

Although it was classically believed that CIA resulted from an aberrant Th1 response, recent data call the role of the Th1 response in the pathogenesis of CIA into question. A deficiency in the Th1-associated genes *Ifng*, *Ifngr* or *Il12p35* was reported to accelerate CIA in mice [9], [21]. *Il23p19* ^−/−^ mice are resistant to the development of CIA, and the inhibition of IL-17 or over-expression of IL-17 in the joints suppresses or worsens joint inflammation, respectively [24], [25]. Th17 and IL-17 appear to be more pathogenic in CIA than Th1 and its related cytokines. However, p35 is also a subunit of IL-35, a regulatory cytokine constitutively expressed by mouse Foxp3^+^ Treg cells [1], [26]. The simultaneous absence of IL-12 and IL-35 in p35^−/−^ mice may mask the pro-inflammatory effects of IL-12. Our data demonstrated that both CD4^+^IFN-*γ* ^+^ T cells and CD4^+^IL-17A^+^ T cells were elevated in CIA control mice, suggesting that both Th1 and Th17 phenotypes may play important roles in the pathogenesis of CIA.

Adult male and female *S. japonicum* worms live in mesenteric veins, where they mate, produce thousands of eggs per day by the female worm [1], [22], [27]. To survive for the long-term in the host, adult worms have exploited certain immune evasion mechanisms. In contrast, the successful excretion of eggs from the intestinal wall to facilitate their life cycles and the development of granulomatous lesions are clearly dependent on CD4^+^ T cells [1], [2], [22]. In addition, the development of the Th2-dominant response coincides with the onset of eggs produced by adult worms [2]. Eggs or soluble egg antigens (SEA) can induce an intense Th2 response even without the presence of adjuvant. Glycoconjugates from egg, such as lacto-N-fucopentaose III (LNFPIII), lysophosphatidylserine, and glycoprotein IPSE (IL-4 inducing principle of *S. mansoni* eggs), can produce potent Th2 response both *in vivo* and *in vitro* [1], [2], [28], [29]. The newest data demonstrated that Omega-1, a T2 ribonuclease glycoprotein from *S. mansoni* eggs, is capable of evoking a Th2 response with similar characteristics as whole SEA [30]. The eggs seem to be more immunogenic and potent to induce Th2 response than worms do. However, the Th2 response was induced during pre-patent infection (e.g., 4 weeks after bisexual *S. masoni* infection), and unisexual *S. masoni* infection also evoked a Th2 response [16], [31], suppressing Th1 dominant diabetes in NOD mice [1], [31]. Consistent with these studies, our results showed that prior to CII immunization, both unisexual and bisexual schistosome infection could reduce the enhancement of CD4^+^IFN-*γ* ^+^ T cells in the spleen. Together with our observations of cytokine profiles and tritiated thymidine incorporation from ConA-stimulated splenocytes, we determined that both worms and eggs can evoke Th2 responses, systemically modulate the expression of pro/anti-inflammatory cytokines and reduce the proliferation of splenocytes. This has been proposed as a major mechanism for the prevention of Th1 mediated autoimmune disease [1], [2], [12], [27], [32], [33].

Despite the successful generation of a Th2 response, the long-term persistence of a parasite within an immunocompetent host is partially facilitated by the induction of Tregs, resulting in an equilibrium that can become mutually beneficial to both the parasite and the host [1], [27], [29], [32], [33]. CD25^+^ Tregs are a subset of CD4^+^ T cells with a critical role in the prevention of autoimmunity [34], [35]. There is already evidence indicating that promoting the function or increasing the numbers of Tregs ameliorates a wide range of experimental arthritis and RA patients [35]. Tregs were identified as a major source for IL-10 in schistosome-infected mice [1], [27]. These CD4^+^CD25^+^ T cells are potent immunosuppressors and are able to inhibit the proliferation of effector T cells both *in vitro* and *in vivo* [17], [35], primarily by limiting Th1 development and promoting a polarized Th2 response [36]. CD4^+^ T cells that express high levels of CD25 are believed to be generally Foxp3^+^ with a high immunosuppressive ability [37]. Thus, we examined the percentage of CD4^+^CD25^high^ T cells and found that this population was markedly elevated and CD4^+^IL-17A^+^ T cells were reduced only in the prior bisexual schistosome-infected mice compared with CIA control mice as measured by FCM, which was further confirmed by an increase in Foxp3 mRNA in the splenocytes. The inconsistent results of IL-17A in FCM and ELISA in our study might be related to the limited stability of this cytokine in the supernatants [25]. This phenomenon is of interest because it suggests that unisexual and bisexual infection modulate the host immune response in different ways. The bisexual schistosome infection not only increased the absolute number of splenocytes but also elevated the percentage of Th2 and CD4^+^CD25^high^ T cells in CIA mice. Thus we inferred that the egg antigen could be more powerful immunogen to the host than the worm antigen, and the egg deposition might be the major stimulus to down-regulate Th17 responses in murine experimental autoimmune arthritis. However, we could not confirm whether the eggs play a key role in Treg and Th17 responses in CIA. To evaluate the effects of eggs on the balance between Treg and Th17 responses, additional investigations on live eggs, SEA or excretory/secretory egg product from *S. japonicum* should be conducted.

In tissue-specific autoimmunity, the balance of cytokines is a key determinant of resistance or susceptibility [21]. The cytokine environment during priming and the consequent activation of specific transcription factors are two key elements regulating the phenotype of effector and regulatory T cells in the chronic inflammatory synovium, which is different from the architecture and cellular constituents of spleen. Thus, we examined the levels of mRNA expression of cytokines and the Th cell lineage-specific transcription factors in the inflammatory joints. Consistent with the observation of cytokine patterns in spleen, the elevated TNF-*α*, IL-1*β* and IL-6 expression in CIA mice were markedly attenuated, whereas IL-10 were increased by prior *S. japonicum* infection. Moreover, the expression of RANKL, an essential differential and activating factor for osteoclast development, was also reduced in prior *S. japonicum* infected CIA mice. The protective mechanism involved in our study might be associated with the enhanced expression of the regulatory cytokine IL-10 in inflammatory sites.

Examining Th cell subpopulations, we noted a decrease in the Th1 and Th17 signature cytokines IFN-*γ* and IL-17A and their specific transcription factors (T-bet and RORγt), while Th2 and Treg specific transcription factors (GATA3 and Foxp3), were elevated in prior infected CIA mice. Although the newest data implied that the IL-17A-producing Th17 lineage appeared to supplant Th1 cells in promoting autoimmunity, our data showed that the Th1 phenotype might also act as pathogenic agents in CIA. Unexpectedly, we did not observe any increase in IL-4 levels in the local inflammatory joints in any experimental animals, which could be explained by a relative paucity of Th2 cells in the inflammatory sites of CIA mice [38]. Due to prolonged exposure to pro-inflammatory cytokines such as TNF-*α* in the inflamed synovium, the phenotype and function of synovial T cells may be quite different from that of lymph tissue and peripheral blood [21], [23]. To determine effects of specific Th lineage and lineage-related cytokines on the inflamed joint, additional experiments on synovium Th cell subpopulations using gene engineering DBA/1 mice may be necessary.

This is the first time that both prophylactic and therapeutic strategies were used to explore the immunoregulatory effects of *S. japonicum* unisexual and bisexual infection on CIA *in vivo*. These results lead to the conclusion that *S. japonicum* infection systemically evoked an immunomodulatory environment and ameliorated autoimmune collagen-induced arthritis in DBA/1 mice. The systemic Th2-dominant response elicited by both unisexual and bisexual infection may involve in suppressing the overactive Th1 response in CIA. Antigens from bisexual infection may be powerful inducers of Tregs, which have been implicated in maintaining a balance between Treg and Th17 responses in autoimmune arthritis. The mechanism of suppression may involve down-regulating the expression of pro-inflammatory cytokines and up-regulating the expression of regulatory cytokines. The beneficial effects of prior *S. japonicum* schistosome infection also included restoring the homeostatic T cell immune responses in local inflamed joints, reducing the activation of osteoclasts and ameliorating the joint destruction. In contrast, post *S. japonicum* infection could not reverse the activated immune response and subsequent lesion.

Helminths and helminth-derived products are apparently well tolerated by their hosts [29], [39]. However, it would be far more desirable would be to mimic the beneficial effects of helminth infection with immunomodulators derived from helminths. Emerging proteomics techniques and the progress in the *S. japonicum* sequencing project will surely bring us closer to identifying and using these molecules as new, safe therapeutic agents to prevent or control the risks inherent in live infection [40], [41]. Using a two-dimensional electrophoresis technique followed by peptide sequencing, our group has cloned and expressed a number of proteins from adult *S. japonicum* that could be responsible for inducing the polarization of the Th2 cell response *in vivo* (data unpublished). Additional work is now underway to develop schistosome-derived immunomodulators with promising potential as anti-inflammatory agents for autoimmune disorders.

## Materials and Methods

### Animals

Male DBA/1 mice (8–10 weeks) were purchased from the Institute of Materia Medica Chinese Academy of Sciences (Shanghai, China). They were housed at the animal facility of the Laboratory Animals Centre of Anhui Medical University. Animals were fed *ad libitum*. This study was carried out in strict accordance with the recommendations in the Guide for the Care and Use of Laboratory Animals of the National Institutes of Health. The protocol was approved by the Institutional Review Board of the Anhui Medical University Institute of Biomedicine (Permit Number: AMU42-050609).

### Mice infected with *S. japonicum*

*Oncomelania hapensis* (strain of Chinese mainland) was purchased from Jiangsu Institute of Parasitic Diseases, Wuxi, China. The mice were infected percutaneously with 25 *S. japonicum* cercariae two weeks prior to CII (Sigma-Aldrich, St. Louis, MO) immunization or at the onset of CIA (4 weeks after CII immunization). For a conventional bisexual infection, mice were challenged transcutaneously with mixed cercariae obtained from snails. For unisexual infection, mice were challenged transcutaneously with cercariae obtained from a single snail previously exposed to a single miracidium. The unisexual infection was confirmed by the absence of eggs in the feces and livers and the morphology of worms collected from the mesenteric veins of the infected animals. At 12 weeks after CII immunization, all mice were sacrificed under anesthesia and paws and spleens were prepared for histopathological examination or a cytokine-based analysis.

### Induction and assessment of arthritis

CIA was induced by an intradermal injection of bovine CII emulsified in Freund's complete adjuvant (Sigma-Aldrich, St. Louis, MO) as described previously. The severity of arthritis was assessed using an established scoring system of 0–4 [20], where 0 = no evidence of erythema and swelling, 1 = erythema and mild swelling confined to the tarsals or ankle joints, 2 = erythema and mild swelling extending from the ankle to the tarsals, 3 = erythema and moderate swelling extending from the ankle to metatarsal joints, 4 = erythema and severe swelling encompass the ankle, foot and digits, or ankylosis of the limb. Mice were considered to have arthritis if the arthritis score was more than two points.

### Measurement of anti-collagen IgG in the sera by ELISA

Microplates were coated with 3 µg/ml CII overnight, blocked with 2% bovine serum albumin (Sigma-Aldrich, St. Louis, MO) and then incubated with the sera at a dilution of 1∶1000. Bound total IgG, IgG1, or IgG2a were detected by incubation with HRP-conjugated anti-mouse IgG, IgG1 or IgG2a (Southern Biotech, Birmingham, AL) at 37°C for 1 hrs. Then the plates were washed with PBST and developed with TMB substrate. The reaction was terminated with 4.5N H_2_SO_4_. Optical density (OD) values were measured at 450 nm using an Automatic Microplate Reader (BLx808, BIO-TEK, Winooski, Vermont). The concentration of IgG antibody and its subclasses were calculated by a standard curve prepared by anti-mouse C II IgG(abcam, Hong Kong, China), anti-mouse C II IgG1(Merck, Darmstadt, Germany) or anti-mouse C II IgG2a(abcam, Hong Kong, China) respectively.

### Quantitation of cytokine production

At 12 weeks after CII immunization, the spleen was removed aseptically. A single cell suspension was prepared by density gradient centrifugation using Mouse Lymphocyte Separation Medium (Solarbio, Beijing, China). Splenocytes were then cultured in RPMI 1640 containing 10% fetal calf serum (FCS; HyClone, Logan, Utah), 2×10^−5^ M 2-mercaptoethanol, and 20 mM L-glutamine at a density of 5×10^6^ cells/ml with ConA (5 µg/ml) for 48 hrs. Supernatants were collected and analyzed for cytokine production (TNF-*α*, IFN-*γ*, IL-1*β*, IL-2, IL-4, IL-6, IL-10 and IL-17A) by ELISA according to the supplier's instructions (R&D Systems, Minneapolis, MN).

### Analysis of splenocyte proliferation by tritiated thymidine incorporation test

For *in vitro* proliferation studies, at 12 weeks after CII immunization, splenocytes (5×10^6^ cells/ml) were cultured in 200 µl RPMI 1640 containing 10% FCS with ConA (5 µg/ml) and CII (50 µg/ml or 100 µg/ml) for 72 hrs in a 96-well plate. During the last 16 hrs of culture, cells were pulsed with 1 µCi of tritiated thymidine and then harvested and counted in a scintillation counter (LS6500, Bekman, Brea, CA).

### Analysis of CD4^+^ T cell subpopulations by flow cytometry

For Treg analysis, splenocytes were extracellularly stained with FITC-conjugated anti-CD4 and PE-conjugated anti-CD25 (eBioscience, San Diego, CA). The CD25^high^ population was defined based on a CD25 mean fluorescence intensity (MFI) two-fold higher than that of CD4^+^CD25^+^ T cells. For all intracellular cytokine staining, splenocytes were stimulated with PMA/ionomycin in the presence of monesin (Alexis, Farmingdale, NY) for 4 hrs. Cells were harvested, extracellularly stained with FITC-conjugated anti-CD4, fixed and permeabilized with IntraPrep™ Permeabilization Reagent (Beckman Coulter, Marseille, France), and then intracellularly stained with PE-conjugated anti-IFN-*γ*, anti-IL-4 and anti-IL-17A (eBioscience, San Diego, CA) according to the manufacturer's recommendations. Data were acquired and analyzed on an XL-EPICS MCL with system II (Beckman Coulter, Brea, CA). Live events were collected based on forward and side scatter patterns. The absolute number of Th subpopulations was calculated as: the percentage of Th subset×total number of splenocytes.

### Quantification of cytokine mRNA by real-time PCR

RNA from the paws or splenocytes was isolated with TRIzol (Invitrogen, Carlsbad, CA). RNA was converted to cDNA using the Omniscript reverse-transcription kit (Promega, Madison, WI) according to the manufacturer's protocol. Primers and TaqMan probes were synthesized using an Applied Biosystems synthesizer and gene expression assay probes at ShineGene (Shanghai, China). The primers/probe assay IDs are AA14879 (TNF-*α*), AA14882 (IFN-*γ*), ASA07075 (IL-1*β*), AA14891 (IL-4), ASA07072 (IL-6), AA14897 (IL-10), AA14900 (IL-17A), AA14903 (T-bet), AA14906 (GATA3), AA14912 (Foxp3), AA14909 (RORγt), AA14915 (RANKL) and AA14918 (*β*-actin). An ABI prism 7500 sequence detection system (Applied Biosystems, Foster City, CA) was used for 40 cycles of PCR. cDNA and Premix Ex Taq™ (Takara, Dalian, China) were used for the amplification of mouse cytokine and RANKL cDNA. The thermal cycling condition was programmed according to the manufacturer's instructions. The cytokines mRNA expression levels were normalized against *β*-actin (endogenous control). Five serial dilutions of cDNA pooled from each sample were used to create a standard curve against which *β*-actin and cytokine mRNA expression were normalized.

### Histopathologic analysis

Hind paws were fixed for 48 hours in 10% buffered formalin and decalcified in 15% EDTA. The paws were then embedded in paraffin, and serial 4 µm sagittal sections of whole right keens were cut and stained with hematoxylin and eosin (HE).

### Statistical analysis

All data are expressed as mean ± SD. Unless otherwise noted, combined experimental data were analyzed by one-way ANOVA with Bonferroni's multiple comparison tests. Intergroup survivals were compared by log-rank test of Kaplan–Meier survival curves. All statistical analyses were performed using GraphPad Prism software. *P*<0.05 was considered statistically significance.
